# The Pulmonary Endothelial Glycocalyx Modifications in Glypican 1 Knockout Mice Do Not Affect Lung Endothelial Function in Physiological Conditions

**DOI:** 10.3390/ijms241914568

**Published:** 2023-09-26

**Authors:** Lakshmi N. R. Thota, Joaquin E. Lopez Rosales, Ivan Placencia, Evgeny A. Zemskov, Paola Tonino, Ashley N. Michael, Stephen M. Black, Andreia Z. Chignalia

**Affiliations:** 1 Department of Anesthesiology, College of Medicine-Tucson, The University of Arizona, Tucson, AZ 85724, USAlopezjoaquin775@arizona.edu (J.E.L.R.);; 2 Center for Translational Science, Florida International University, Port St. Lucie, FL 34987, USA; 3 Department of Cellular Biology & Pharmacology, Howard Wertheim College of Medicine, Florida International University, Miami, FL 33199, USA; 4 Research, Innovation & Impact Cores Facilities, Imaging Cores-Electron, Life Sciences North, The University of Arizona, Tucson, AZ 85719, USA; toninop@arizona.edu; 5 Asthma and Airway Disease Research Center, The University of Arizona, Tucson, AZ 85724, USA; 6 Department of Environmental Health Sciences, Robert Stempel College of Public Health and Social Work, Florida International University, Miami, FL 33174, USA; 7 Department of Physiology, College of Medicine-Tucson, The University of Arizona, Tucson, AZ 85724, USA; 8 Sarver Heart Center, The University of Arizona, Tucson, AZ 85724, USA; 9 Department of Pharmacology and Toxicology, College of Pharmacy, The University of Arizona, Tucson, AZ 85721, USA

**Keywords:** glypican 1, glycocalyx, lung, heparan sulfate, endothelial, eNOS

## Abstract

The endothelial glycocalyx is a dynamic signaling surface layer that is involved in the maintenance of cellular homeostasis. The glycocalyx has a very diverse composition, with glycoproteins, proteoglycans, and glycosaminoglycans interacting with each other to form a mesh-like structure. Due to its highly interactive nature, little is known about the relative contribution of each glycocalyx constituent to its overall function. Investigating the individual roles of the glycocalyx components to cellular functions and system physiology is challenging, as the genetic manipulation of animals that target specific glycocalyx components may result in the development of a modified glycocalyx. Thus, it is crucial that genetically modified animal models for glycocalyx components are characterized and validated before the development of mechanistic studies. Among the glycocalyx components, glypican 1, which acts through eNOS-dependent mechanisms, has recently emerged as a player in cardiovascular diseases. Whether glypican 1 regulates eNOS in physiological conditions is unclear. Herein, we assessed how the deletion of glypican 1 affects the development of the pulmonary endothelial glycocalyx and the impact on eNOS activity and endothelial function. Male and female 5–9-week-old wild-type and glypican 1 knockout mice were used. Transmission electron microscopy, immunofluorescence, and immunoblotting assessed the glycocalyx structure and composition. eNOS activation and content were assessed by immunoblotting; nitric oxide production was assessed by the Griess reaction. The pulmonary phenotype was evaluated by histological signs of lung injury, in vivo measurement of lung mechanics, and pulmonary ventilation. Glypican 1 knockout mice showed a modified glycocalyx with increased glycocalyx thickness and heparan sulfate content and decreased expression of syndecan 4. These alterations were associated with decreased phosphorylation of eNOS at S1177. The production of nitric oxides was not affected by the deletion of glypican 1, and the endothelial barrier was preserved in glypican 1 knockout mice. Pulmonary compliance was decreased, and pulmonary ventilation was unaltered in glypican 1 knockout mice. Collectively, these data indicate that the deletion of glypican 1 may result in the modification of the glycocalyx without affecting basal lung endothelial function, validating this mouse model as a tool for mechanistic studies that investigate the role of glypican 1 in lung endothelial function.

## 1. Introduction

The glycocalyx is a cellular surface layer ubiquitously expressed in all cells [1]. This cellular coat was first observed in endothelial cells, where it was described to be a physical barrier to the passage of solutes and proteins from the circulation to the interstitium [2,3]. It is currently known that the glycocalyx is a dynamic signaling layer that is involved in cellular homeostasis and pathological processes such as inflammation [4], hyperpermeability [5,6,7], coagulation [8,9], and leukocyte migration [5,10,11]. Moreover, degradation of the glycocalyx is often seen during disease development [12,13,14] and thus, the glycocalyx is seen as a gatekeeper for cellular homeostasis.

The glycocalyx shows mobile characteristics that result from its chemical composition. This unique cellular coat is assembled and stabilized by strong covalent interactions between glycoproteins, proteoglycans, and glycosaminoglycans. The high mobility of this layer allows the interaction between individual components of the glycocalyx with multiple membrane and matrix proteins as well as circulating factors [15]. Due to this extremely interactive property, the cellular and physiological functions attributed to the glycocalyx have been interpreted as the result of its activation as a unit [16]. Recently, studies have focused on trying to understand the individual contribution of glycocalyx components to its overall function [17,18,19,20,21,22].

The investigation of individual roles of the glycocalyx components to cellular functions and system physiology is challenging, as it faces technical limitations including the lack of specific pharmacological inhibitors and adaptive responses to the use of molecular tools such as siRNA, shRNA, and gene editing. Such adaptive responses include the disruption of glycocalyx stability and the compensatory expression of specific components in an attempt to maintain glycocalyx function. Thus, it is crucial that genetically modified animal models for glycocalyx components are characterized and validated before the development of mechanistic studies.

Among the glycocalyx components, membrane-bound heparan sulfate proteoglycans (HSPGs) have received particular attention, as they are considered the backbone of the glycocalyx and potential candidates for signal transduction. Mainly, glypican 1 has stood out as a regulator of cardiovascular function. Recent studies have shown that glypican 1 knockout mice (*Gpc1*^−/−^) show reduced systemic blood pressure [18] and changes in the electrical properties of the heart [23]. These mice also have impaired endothelium-dependent vasorelaxation and decreased flow-induced activation of endothelial nitric oxide synthase (eNOS) [18,24]. Interestingly, the expression of glypican 1 is decreased in older mice, and this has been discussed as a potential mechanism for the development of vascular disease [25].

The main effects of glypican 1 in the cardiovascular system have been associated with the impaired activation of eNOS and decreased nitric oxide (NO) production. This has been observed in response to shear stress [26,27] or matrix stiffness [28] in vitro. This has also been observed as an impaired response to acetylcholine (ACh) in isolated aortic rings in glypican 1 global knockout mice ex vivo [18]. In the pulmonary vasculature, eNOS is known to regulate endothelial barrier function during disease states. However, the effects of glypican 1 on eNOS signaling in the lungs and pulmonary homeostasis have not been explored. Herein, we sought to characterize the impact of the global deletion of glypican 1 on overall pulmonary physiology.

## 2. Results

### 2.1. Global Deletion of Gpc1 Leads to Increased Glycocalyx Thickness in the Lung Vasculature

**Figure 1:**
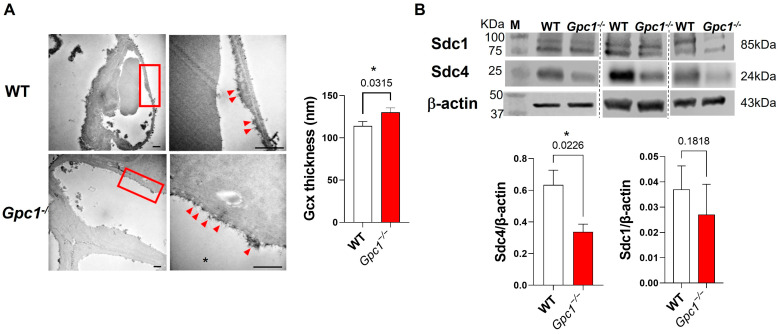
**Glypican 1 knockout mice show a modified glycocalyx.** (**A**) Representative electron micrographs of WT and *Gpc1*^−/−^ showing endothelial glycocalyx (left panel), areas selected in the red square are shown at higher magnification on the right panel with prominent endothelial glycocalyx cluster-like pattern (red arrowheads). In the plot, *Gpc1*^−/−^ showed increased endothelial glycocalyx thickness when compared to WT. Scale bar: 0.5 µm. N = 190 measurements for WT and N = 230 measurements for *Gpc1*^−/−^. (**B**) *Gpc1^−/−^* showed decreased expression of syndecan 4 (Sdc4) but not of syndecan 1 (Sdc1) in lung tissue when compared to WT; *p* values resulting from unpaired Student’s *t* test are shown for each comparison. * Indicates statistical significance (*p* < 0.05) vs. WT, unpaired *t* test, N ≥ 5/group. Representative bands from different gels or not in consecutive sequence are separated by dashed lines. Brightness and contrast adjustments were applied equally to all images for representative images. M: molecular weight ladder.

To assess if the global deletion of the *Gpc1* gene changed the glycocalyx tertiary structure in pulmonary vessels, WT and *Gpc1*^−/−^ lung sections were imaged by transmission electron microscopy. Global deletion of glypican 1 resulted in the increased thickness of the lung endothelial glycocalyx in comparison to wild-type lungs (114 ± 5.26 in WT vs. 130 ± 5.22 in *Gpc1*^−/−^). In addition, *Gpc1*^−/−^ showed a different distribution of the glycocalyx in endothelial surfaces, with a cluster-like pattern in which the glycocalyx branches were elongated (Figure 1A).

### 2.2. Global Deletion of Gpc1 Modified the Chemical Composition of the Glycocalyx

**Figure 2:**
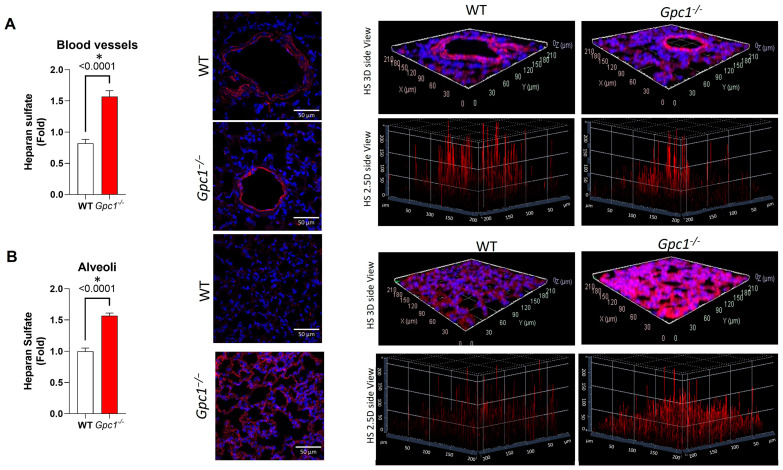
**Glypican 1 knockout mice show increased heparan sulfate content in the lungs.***Gpc1*^−/−^ lungs showed higher heparan sulfate staining in (**A**) blood vessels and (**B**) alveolar compartment; White Bar indicates 50 µm; *p* values resulting from unpaired Student’s *t* test are shown for each comparison. * Indicates statistical significance (*p* < 0.05) vs. WT, unpaired *t* test. N = 19 measurements for WT and N = 20 measurements for *Gpc1*^−/−^.

To assess if the global deletion of Gpc1 changes the chemical composition of the glycocalyx, we assessed the total expression of syndecan 1, syndecan 4, and heparan sulfate, as they are major components of the endothelial glycocalyx. *Gpc1*^−/−^ mice showed an approximate 50% decrease in the expression of syndecan 4 when compared to WT mice, as assessed by immunoblotting in lung lysates (Figure 1B). A trend towards a decrease in syndecan 1 was observed. However, no statistically significant difference in syndecan 1 expression was found between *Gpc1*^−/−^ and WT lung lysates (Figure 1B). Total heparan sulfate content was assessed by immunofluorescence in lung sections of WT and *Gpc1*^−/−^ mice. Total heparan sulfate content almost doubled in pulmonary blood vessels of *Gpc1*^−/−^ when compared to WT lungs (Figure 2A) and was increased by about 50% in the distal part of the lungs (alveoli) (Figure 2B).

### 2.3. Glypican 1 Knockout Mice Show Decreased eNOS Activity with Preserved Nitric Oxide Production

**Figure 3:**
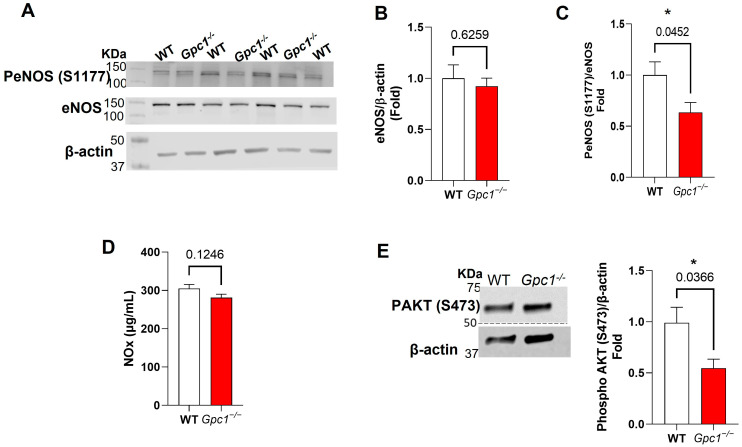
**Decreased eNOS activity in glypican 1 knockout lungs does not affect NOx production.** (**A**,**B**) eNOS total expression is not altered in *Gpc1*^−/−^ lungs when compared to WT. (**A**,**C**) *Gpc1*^−/−^ lungs showed decreased eNOS activation compared to WT lungs as assessed by the phospho S1177 eNOS/total eNOS ratio. (**D**) Nitric oxide production is not changed in *Gpc1*^−/−^ lungs. (**E**) The phosphorylation of AKT at S473 is decreased in *Gpc1*^−/−^ mice; *p* values are shown for each comparison. * Indicates statistical significance (*p* < 0.05) vs. WT, N ≥ 5/group. Unpaired Student’s *t* test.

As eNOS has been described as a primary target for glypican 1 in the cardiovascular system, we assessed whether the global deletion of *Gpc1* affects eNOS activation in the lungs in physiological conditions. The expression of total eNOS and its phosphorylation at S1177 was assessed in WT and *Gpc1*^−/−^ lung lysates. No differences were observed in total eNOS expression between mouse strains. *Gpc1*^−/−^ mice showed an approximate 40% decrease in eNOS activation when compared to the WT as assessed by the phosphorylation of eNOS at S1177 (Figure 3A–C). To assess if the decreased activation of eNOS seen in *Gpc1*^−/−^ affected nitric oxide (NOx) production, NOx levels were assessed in total lung lysates. No differences in NOx levels were observed between groups (281.3 ± 8.9 µg/mL vs. 304.9 ± 10.46 µg/mL in WT) (Figure 3D). To determine the mechanism whereby glypican 1 decreases the phosphorylation of eNOS at S1177, we assessed if the phosphorylation of AKT at Ser 473, a positive regulator of eNOS, was altered in *Gpc1*^−/−^ mice. *Gpc1*^−/−^ lungs showed a significant decrease in the phosphorylation of eNOS at S473 when compared to the WT (0.54 ± 0.08 in *Gpc1*^−/−^ vs. 0.98 ± 0.15 in WT) (Figure 3E).

### 2.4. Decreased eNOS Activity Does Not Affect Endothelial Barrier Formation In Vitro

**Figure 4:**
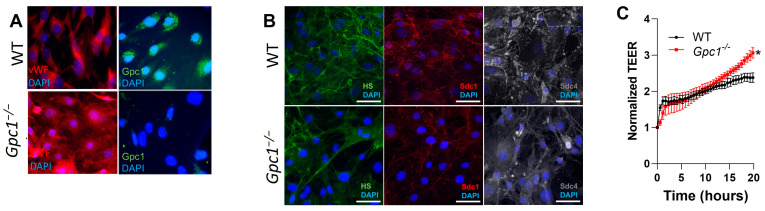
*Gpc1*^−/−^ MLECs form monolayers with increased resistance in vitro. (**A**) The primary isolation of MLECs is pure, with all cells staining for von Willebrand factor, a marker of endothelial cells. The glypican 1 knockout mice are validated with no expression of glypican 1 in primarily isolated MLECs. Images shown at 40× magnification. (**B**) MLECs show glycocalyx in vitro as evidenced by the staining of heparan sulfate (HS), syndecan 1 (Sdc1), and syndecan 4 (sdc4) in both WT and *Gpc1*^−/−^ MLECs. White bar length: 50 µm. (**C**) *Gpc1*^−/−^ MLECs form a tighter endothelial barrier in vitro, as shown by increased TEER when compared to WT MLECs. * Indicates statistical significance (*p* < 0.05) vs. WT, N ≥ 3/group, two-way ANOVA.

eNOS is a known regulator of the pulmonary endothelial barrier. To assess if the deletion of *Gpc1*^−/−^ affects the lung endothelial barrier, we isolated MLECs from WT and *Gpc1*^−/−^ lungs. Figure 4A shows that our primary cell culture is pure, as all cells show staining for von Willebrand factors, a marker of endothelial cells. Moreover, we show that glypican 1 is not present in cells isolated from *Gpc1*^−/−^, validating the knockout model. We then confirmed that our primarily isolated MLEC forms the glycocalyx in vitro. Figure 4B shows that MLECs from WT and *Gpc1*^−/−^ mice express heparan sulfate, syndecan 1, and syndecan 4, all major endothelial glycocalyx components, when cultured in vitro. To assess if the deletion of glypican 1 affects endothelial barrier formation in vitro, MLECs were plated in gold electrodes, and TEER was measured in real time. *Gpc1*^−/−^ MLECs showed increased TEER compared to WT MLECs (3.06 ± 0.14 in *Gpc1*^−/−^ vs. 2.38 ± 0.13 in WT) (Figure 4C).

### 2.5. Pulmonary Ventilation and Mechanics in Glypican 1 Knockout Mice

**Figure 5:**
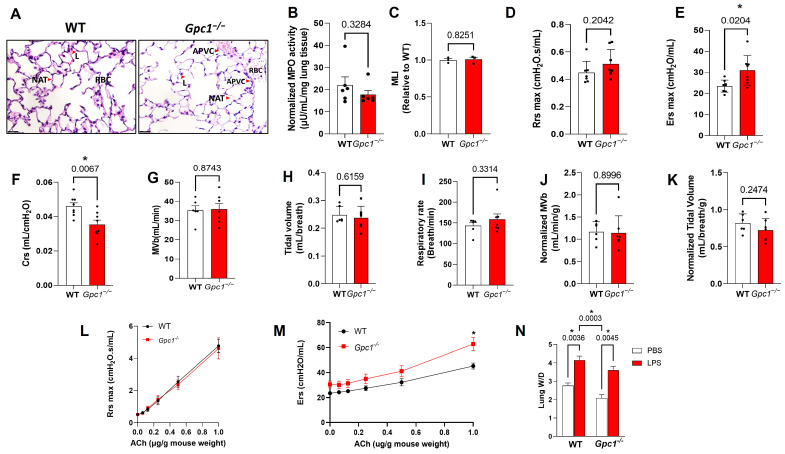
**The airway phenotype of glypican 1 knockout mice.** (**A**) *Gpc1*^−/−^ lungs show no evidence of lung injury (perivascular cuffing, fluid extravasation to alveolar compartment, cellular infiltration, or protein exudate). Red arrows indicate APVC (absence of perivascular cuffing), NAT: normal alveolar wall thickness; RBS: red blood cells; L: leukocyte. Black bar indicates 25 µm. (**B**) *Gpc1*^−/−^ shows no changes in lung MPO activity. (**C**) *Gpc1*^−/−^ shows no changes in lung alveolar surface area. *Gpc1*^−/−^ shows (**D**) preserved airway resistance, (**E**) increased lung elastance, and (**F**) decreased lung compliance when compared to WT mice. *Gpc1*^−/−^ shows (**G**) similar minute ventilation, (**H**) tidal volume, and (**I**) respiratory rate when compared to WT mice. (**J**,**K**) Normalization of minute ventilation and tidal volume do not indicate significative differences between mouse strains. (**L**,**M**) The airway mechanics follow physiological phenotype when challenged with ACh: *Gpc1*^−/−^ shows similar resistance and increased elastance. (**N**) *Gpc1*^−/−^ develops pulmonary edema in the same magnitude as the WT mice; *p* values resulting from unpaired Student’s *t* test (graphs (**B**–**K**)) and two-way ANOVA (**L**–**N**)) are shown. * Indicates statistical significance (*p* < 0.05) vs. WT: N = 3 for (**A**,**C**); N ≥ 4/group for N; and N ≥ 5 for (**B**,**D**–**M**).

As glypican 1 is expressed in endothelial and epithelial pulmonary cells, we assessed if the glypican 1 global knockout showed differences in overall lung structure by its histological features. The histological analysis of *Gpc1*^−/−^ lungs showed no major changes in airway structure, as evidenced by the similar alveolar surface area found between mice strains as assessed by the MLI. In addition, no evidence of lung injury was seen, as histological markers of lung injury (alveolar wall thickening, perivascular cuffing, and protein/cellular infiltrates) were not observed in *Gpc1*^−/−^ lung sections. We additionally assessed myeloperoxidase (MPO) activity as indices of leukocyte recruitment to the lungs and inflammation. Glypican 1 knockout lungs showed similar MPO activity levels as wild-type lungs (17.7 ± 1.87 in *Gpc1*^−/−^ vs. 22.03 ± 3.69 in WT), confirming the absence of inflammation and leukocyte migration in the *Gpc1*^−/−^ lungs (Figure 5A–C).

We then assessed pulmonary mechanics using the flexivent system. *Gpc1*^−/−^ mice showed preserved airway resistance (0.51 ± 0.04 in *Gpc1*^−/−^ vs. 0.44 ± 0.03 in WT) but increased lung elastance (30.92 ± 2.48 in *Gpc1*^−/−^ vs. 23.43 ± 1.02 in WT) when compared to WT mice. Compliance, the reciprocal of elastance, was decreased in *Gpc1*^−/−^ mice (0.035 ± 0.002 in *Gpc1*^−/−^ vs. 0.046 ± 0.002 in WT) (Figure 5B–F). We further assessed pulmonary function by analyzing the ventilation in awake, non-anesthetized WT and *Gpc1*^−/−^ mice. There were no differences in minute volume (MVb, 34.18 ± 8.54 in WT vs. 35.00 ± 11.1 in *Gpc1*^−/−^), tidal volume (VT) (0.24 ± 0.03 µL in WT vs. 0.21 ± 0.033 µL in *Gpc1*^−/−^), and respiratory rate (f; 139.1 ± 26.48 in WT vs. 168.5 ± 54.31 in *Gpc1*^−/−^) between mouse strains (Figure 5G–I). Ventilation parameters corrected by animal weight are also shown (Figure 5J,K). To assess if decreased eNOS phosphorylation at S1177 could decrease airway responsiveness to ACh, a known NOS regulator and a mediator of allergic reactions processed in the lungs [29], we assessed lung resistance and elastance in response to ACh. Lung mechanics followed the baseline phenotype, as ACh had no effect on lung resistance in both WT and *Gpc1*^−/−^ lungs, but the elastance response to ACh was increased in *Gpc1*^−/−^ lungs (32.206 ± 2.88 in WT vs. 41.188 ± 4.5 in *Gpc1*^−/−^ at the 0.5 mg/g dose). To test if *Gpc1*^−/−^ lungs showed different susceptibility to a sepsis model of acute lung injury (ALI), we assessed their susceptibility to develop pulmonary edema, a known outcome of acute lung injury and ARDS, using sepsis model, in which mice were injected with LPS 10 mg/Kg, intraperitoneally. Both WT and *Gpc1*^−/−^ lungs developed lung edema 6 h after LPS injection (3.606 ± 0.2130 in *Gpc1*^−/−^LPS and 4.16 ± 0.22 in WT-LPS) (Figure 5L–N).

## 3. Discussion

The glycocalyx is a surface layer known to be degraded during pathological processes. In the lung vasculature, endothelial glycocalyx degradation has been associated with endothelial hyperpermeability and the development of pulmonary edema in conditions of increased hydrostatic pressure [30,31]. Therefore, the endothelial glycocalyx is considered a protective structure in the lung vasculature.

The diverse and highly interactive nature of the glycocalyx gives this surface layer a putative unison signaling mechanism, in which chemical or physical stimuli are thought to produce a single response attributed to the overall activation or disturbance of the glycocalyx. Due to its complex tertiary structure, the knowledge of the relative contribution of each glycocalyx component to the protective effects of the glycocalyx on endothelial function is unexplored.

The paucity of studies addressing specific roles of glycocalyx components on cellular function results, at least in part, from technical limitations such as the lack of specific pharmacological inhibitors and changes in the chemical composition of the glycocalyx when molecular tools are used. Although changes in the chemical composition of the glycocalyx are known to occur secondary to the genetic deletion of specific components, there is often a lack of assessment of how such changes impact a specific cellular function/property.

In this study, we sought to explore how the genetic deletion of glypican 1 altered the chemical composition of the glycocalyx and whether such changes could affect the lung endothelial barrier and overall pulmonary function to validate the use of global glypican 1 knockout mice to the study of pulmonary diseases and acute lung injury. The main finding of this study is that the global deletion of glypican 1 does alter the chemical composition of the glycocalyx without impairing overall pulmonary function as assessed by endothelial barrier stability and pulmonary ventilation parameters, suggesting that global knockout mice for glypican 1 can be used as a model for the investigation of the individual role of glypican 1 in pulmonary vascular diseases.

Glypican 1 knockout mice showed increased thickness of the pulmonary endothelial glycocalyx compared to wild-type mice. Interestingly, *Gpc1*^−/−^ lungs showed a clustered-like pattern of the glycocalyx in association with elongated glycocalyx filaments, which suggested changes in the chemical composition of the lung endothelial glycocalyx. Indeed, we observed increased total heparan sulfate content and the decreased expression of syndecan 1 and syndecan 4, the other types of membrane-bound HSPGs expressed in endothelial cells [32]. These findings suggest that the global deletion of glypican 1 may be a glycocalyx “stressor” impacting the stability of the backbone of the glycocalyx that, in turn, activates compensatory mechanisms to maintain the cellular surface layer, such as the increase in non-membrane-bound HSPG content in the lungs, resulting in the increased glycocalyx thickness observed in these mice.

Changes in glycocalyx dimensions are often associated with pathological processes. A decreased glycocalyx was observed at arterial branch points, prone to the development of atherogenic lesions [33]. Additionally, glycocalyx perturbation is associated with impaired endothelial mechanotransduction [34,35], adhesion of platelets [36], and leukocytes [37] to the endothelial surface and endothelial hyperpermeability [7,38,39]. Therefore, we decided to explore if the structural and chemical changes observed in the lung endothelial glycocalyx of glypican 1 knockout mice were associated with lung endothelial barrier disruption, a known process in the early stages of pulmonary vascular diseases.

Among the many factors that regulate lung endothelial barrier stability, eNOS-dependent mechanisms stand out as a common pathway for the glycocalyx effects on lung endothelial barrier function and the effects of glypican 1 on aortic endothelial function. eNOS was shown to be a target of the glycocalyx in conditions of increased pulmonary pressure [7,31,40] and shear stress [26]. Although no studies have reported a link between glypican 1 and eNOS in the lungs, reduced eNOS phosphorylation at S1177 has been associated with decreased glypican 1 in aortic vessels of aged mice [25]. Further, previous studies assessing the role of glypican 1 in aortic vessels have shown decreased expression of eNOS in the descending aorta [18,25] as well as decreased eNOS activity and the production of nitrogen oxide (NOx) [18] in glypican 1 global knockout mice. In aortic rings, the impaired production of NOx was associated with impaired endothelium-dependent vasodilation in basal conditions, an eNOS-dependent effect. Our findings do not match precisely with these prior studies. We show that eNOS activation, as assessed by the phosphorylation of S1177, is decreased in lung homogenates of glypican 1 knockout mice, and this is associated with the decreased phosphorylation of AKT at S473, a known positive regulator of eNOS. However, this decrease in eNOS activity had no impact on the production of NOx, suggesting that glypican 1 knockout mice have preserved lung endothelial function. Although previous studies have shown that glypican 1 is associated with the activation of AKT in cancer cells [41,42], this is the first study to report AKT as a link between glypican 1 and eNOS in the lungs in physiological conditions. To test if the endothelial barrier was preserved after the deletion of glypican 1, we assessed endothelial barrier formation in vitro using MLECs isolated from WT and *Gpc1*^−/−^ mice. *Gpc1*^−/−^ MLECs showed increased transendothelial electrical resistance 24 h after plating compared to WT MLECs. This suggested that the endothelial barrier is preserved in these mice and may form tighter endothelial cell junctions. These findings suggest that lung endothelial function is preserved in glypican 1 knockout mice.

As the endothelial and epithelial barriers of the distal airways are adjacent to each other and glypican 1 is also expressed in epithelial cells, we questioned whether the deletion of glypican 1 impaired the lung airways and pulmonary ventilation. We observed that glypican 1 knockout mice showed a preserved alveolar surface area, no histological signs of lung injury, no changes in MPO activity, and preserved ventilatory parameters (respiratory rate, tidal volume, and minute ventilation). Many studies have implicated the degradation of the glycocalyx in the pathogenesis of lung injury in conditions of trauma, sepsis, or infection [43,44]. Our data suggest that the genetic deletion of glypican 1 in mice does not result in lung injury or changes in basal lung vascular function. This is supported by the lack of pulmonary inflammation as evidenced by levels of myeloperoxidase activity that match lungs from control mice. We found that *Gpc1*^−/−^ lung mechanics are altered with increased elastance and decreased compliance, which is associated with the increased heparan sulfate content. Although lung mechanics are altered in the *Gpc1*^−/−^ mice, their response to LPS, as assessed by lung edema development, does not differ from the WT mice. Whether *Gpc1*^−/−^ mice respond differently to other models of acute lung injury, such as mechanical ventilation, is still not known, and additional studies are required to address such questions. Taken together, our findings indicate that the global deletion of glypican 1 causes changes in the glycocalyx but does not affect pulmonary endothelial function and that this model can be used to study pulmonary vascular disease. In this case, the potential interfering effects from changes in lung mechanics should be considered when assessing outcomes.

## 4. Materials and Methods

### 4.1. Chemicals

Chemicals and reagents were purchased at the highest grade available. Antibodies were as follows: anti-syndecan 1 (Life Technologies, Carlsbad, CA, USA, #362900); anti-syndecan 4 (Santa Cruz Biotechnology, Dallas, TX, USA, #Sc-12766); anti-eNOS (BD Transduction Laboratories, Franklin Lakes, NJ, USA, #2610297); anti phosphoS1177 eNOS (Invitrogen, Carlsbad, CA, USA, # MA5-14957); anti β-actin (Cell Signaling Technologies, Boston, USA, #4967S); anti-heparan sulfate (Amsbio, Cambridge, MA, USA, #370255-S); anti-Tie 2 (Invitrogen, #14-5987-82); anti-von-Willebrand (Abcam, Boston, USA #ab11713); anti-glypican 1 (Invitrogen, #PA5-28055); anti-P-S473 Akt (Santa Cruz, sc-7985-R); Alexa Fluor (Thermo Fisher Scientific, Waltham, MA, USA, #A-21245; #A-11001; A-21236)**;** paraformaldehyde (Thermo Fisher Scientific, Waltham, MA, USA #28908); Dynabeads Sheep anti-Rat IgG (Invitrogen, 11035); glutaraldehyde (Electron Science Microscopy, Hatfield, USA; #16220); sucrose (Sigma, #S-0389); lanthanum nitrate hexahydrate (Electron Science Microscopy, Hatfield, PA, USA; 317300); Cacodylate Buffer (Electron Science Microscopy, Hatfield, PA, USA;11653); normal goat serum (Gibco, Waltham, MA, USA, #16210-064); prolong antifade mounting media (Invitrogen, #P36970); hematoxylin and eosin kit (Vector Laboratories, Newark, CA, USA, #H-3502); ketamine (Covetrus, Phoenix, USA, 11695-0703-1); xylazine (AKORN, Lake Forest, IL, USA, 59399-100-20); Phosphate Buffer Saline (Gibco, 14190-144); Tween 20 (Sigma, Pi379-100 ML); osmium tetroxide (Electron Microscopy Sciences, 19193); Krebs Henseleit solution (mmol/L) (NaCl 130.00, NaHCO_3_ 14.9, C_6_H_12_O_6_ 5.5, KCl 4.7, KH_2_PO_4_ 1.18, MgSO_4_ 1.17, CaCl_2_ 1.6, and HEPES 10.0; pH 7.4); Griess Reagent (Fisher Scientific, Waltham, MA, USA 502009067); sodium nitrite (Fisher Chemical, Pittsburg, PA, USA, 5347-250); Dulbecco’s Modified Eagle Medium (Gibco, 11995-065); Fetal Bovine Serum (FBS) (Corning, MT35011CV); penicillin/streptomycin (Gibco, 15140-122); collagenase 1 (Gibco, #17100-017); Vasculife^®^ VEGF-Mv (Lifeline, Oceanside, USA, #LL-0003); and ProLong™ Gold antifade reagent (Invitrogen, P36934).

### 4.2. Animals

Male and female glypican 1 knockout (*Gpc1*^−/−^) and CD1 (wild-type, WT) mice at 5–9 weeks of age were used. WT mice were purchased from Charles River Laboratories. A breeding pair of *Gpc1*^−/−^ mice were kindly provided to us by Dr. Arthur Lander (UC Irvine). Animal experiments were conducted according to the animal protocols 18-491 and 06-036 approved by The University of Arizona Animal Care.

### 4.3. Whole-Body Plethysmography

Pulmonary ventilation was measured in conscious unrestrained mice by whole-body plethysmography as previously described [45]. Mice were subjected to three acclimation periods of thirty minutes in the plethysmography chamber prior to the date of the experiment. On the day of the experiment, mice were kept in the chamber for 30 min prior to the start of recording ventilation parameters. Tidal volume (VT), minute volume (MVb), and respiratory frequency (f) during quiet breathing were estimated in awake, non-anesthetized mice using Buxco^®^ Small Animal Whole-Body Plethysmography. Experiments were conducted by The University of Arizona Phenotyping Core. For all animals, chamber temperature and humidity were maintained at 22 ± 1 °C and 50 ± 1%, respectively. Ventilation parameters were measured using the Fine Point software V. 2.3.1.9 (Buxco Research Systems, Wilmington, NC, USA). Data are also shown normalized by animal weight.

### 4.4. Pulmonary Mechanics

Pulmonary mechanics were assessed in WT and *Gpc1*^−/−^ mice using the flexivent system (FlexiVent, SCIREQ, Quebec, QC, Canada) as previously described [46]. Briefly, the animals were anesthetized with ketamine/xylazine (100/10 mg/kg, i.p.). The depth of anesthesia was assessed by toe-pinch reflex and signs of heavy breathing. Heart rate was monitored through the procedure. A tracheostomy was performed, and mice were artificially ventilated (10 mL/kg tidal volume, 150 breaths/min, and 3 cmH_2_O PEEP). An intraperitoneal injection of pancuronium bromide induced neuromuscular blockade. Assessments of respiratory mechanics were performed using a 3 s multifrequency volume perturbation. Rrs max (maximal airway resistance), Ers max (maximal elastance), and Crs (lung compliance) were evaluated. To investigate airway responsiveness to ACh, a dose–response curve to Ach (0.1–1 µg/g) was performed. Data were analyzed using Flexiware software (SCIREQ, Montreal, QC, Canada) V.8.3 and are reported as mean ± Standard Error of the Mean (SEM).

### 4.5. Mean Linear Intercept

The mean linear intercept (MLI) was determined as previously described [47]. Briefly, lungs were fixed with 4% PFA and embedded in paraffin. After processing, lung sections were stained with hematoxylin and eosin. Ten lines of a same known length were crossed in a microscope field of a lung section. Every time the transverse crossed an alveolar wall, an intercept was counted. The number of intercepts were counted in both vertical and horizontal lines. The MLI was calculated by using the following formula:

MLI = N × L/m

in which L is the length of the transverses, m is the sum of all intercepts, and N is the number of times the transverses are placed on the lung. Data are presented as mean ± SEM.

### 4.6. Transmission Electron Microscopy

The pulmonary capillary endothelial glycocalyx was imaged using electron microscopy as previously described [48]. Briefly, mice were anesthetized with ketamine/xylazine (90/10 mg/Kg), and the lungs were perfused, via the pulmonary artery, with a fixative solution composed of 2% glutaraldehyde, 2% sucrose, and 2% lanthanum nitrate in 0.1 M Cacodylate Buffer (Ph 7.3). The lungs were harvested and cut into small fragments of approximately 1 mm^3^. Fragments were immersed in the fixative solution for two hours and then transferred to PBS overnight. Fragments were post-fixed with 1% osmium tetroxide, dehydrated in a series of increased ethanol concentrations, and infiltrated and embedded in Spurr resin. Ultrathin sections (90 nm) were obtained and stained with uranyl acetate and lead citrate. Digital images were taken on a FEI Tecnai G2 Spirit BT TEM (FEI, Hilsboro, OR, USA) with a side-mounted camera AMT XR41, operated at 100 kV. The glycocalyx thickness was measured on calibrated images with the AMT software V. 602 (Advanced Microscopy Techniques, Woburn, MA, USA) processing and measurement tool. The lung endothelial glycocalyx thickness was determined at randomly selected areas on calibrated images from WT (n = 10) and *Gpc1*^−/−^ (n = 14), using the AMT Image Capture Engine software V602. The length measurement was performed with the linear (point to point) measurement selected tool, positioning the cursor, and clicking at each end of the structure. Then, the luminal surface of the endothelial cell plasma membrane (start point) and the tip of the glycocalyx cluster-like pattern (end point) were selected, and a line was drawn connecting the two points with the measurement label added in nanometers (nm). The number of length measurement entries (WT: N = 190 and *Gpc1*^−/−^: N = 230) were saved and used for the analyses, and the data are presented as the mean ± SEM.

### 4.7. Western Blotting

Syndecan 1 (1:1000), syndecan 4 (1:1000), phospho eNOS (S1177) (1:500), eNOS (1:1000), phospho Ser 473 AKT (1:500) and β-actin (1:1000) expression was assessed in whole-lung lysates. eNOS activation was determined by the p-Ser^1177^/eNOS ratio. Antibodies were diluted in 3% BSA in TBS-T. Immunoblots were performed as previously described [40]; 5%BSA in TBS-T was used as blocking buffer. Signal was detected by chemiluminescence using the Li-COR Fc system (Li-COR, Lincoln, NE, USA) and band intensities were measured using Image Studio software V5.2 (Li-COR, Lincoln, NE, USA). Data are presented as mean ± SEM. Original membranes can be seen at the Supplementary file.

### 4.8. Colorimetric Griess Reaction

Lung fragments from WT and Gpc1^−/−^ mice (*n* = 5 per group) were incubated in a 24-well plate containing Krebs–Henseleit solution (pH 7.4, 37 °C) for 30 min and then stimulated with Ach (10 μM) for 10 min. Then, 50 μL of the Krebs–Henseileit bath solution of each sample was collected and added to 50 μL of the Griess Reagent (a 1:1 dilution of N-(1-Naphthyl) ethylenediamine dihydrochloride 1% in deionized H_2_O and sulfanilamide 1% in H_3_PO_4_ 5%) in a 96-well plate. Sodium nitrite (3 μmol/L to 200 μmol/L) was used as standard. The absorbance was read at 540 nm and the nitrogen oxide (NOx) concentration was determined.

### 4.9. Myeloperoxidase Activity

Lungs from WT and *Gpc1*^−/−^ mice were harvested and flash frozen for posterior analysis. Myeloperoxidase (MPO) activity was measured using a commercial fluorometric kit (Abcam, Waltham, MA, USA) according to the manufacturer’s instructions. Data are shown in µU/mL and normalized by milligrams of lung tissue as previously published [47].

### 4.10. LPS-Induced Lung Edema

Male and female glypican 1 knockout (Gpc1^−/−^) and CD1 (wild-type, WT) mice were injected with equal volumes of PBS or LPS (10 mg/Kg) intraperitoneally. After 6 h, animals were anesthetized with ketamine/xylazine (90/10 mg/Kg). Anesthesia depth was monitored by toe pinch reflex and signs of heavy breathing. The thorax was opened, and the lungs were harvested. Lungs were weighted and set to dry for 24 h at 60 °C. Lung edema was determined by the ratio of lung wet weight by the lung dry weight as previously published [40]. Data are reported by mean ± SEM.

### 4.11. Primary Isolation of Mouse Lung Endothelial Cells

Mouse lung endothelial cells (MLECs) were primarily isolated as previously described [49] with minor modifications. Mice were anesthetized with ketamine/xylazine (90/10 (mg/Kg)) and shaved. The thorax and abdomen were cleaned with 70% ethanol and chlorhexidine three times and the lungs were harvested and kept in isolating media buffer (DMEM + 20% FBS + penicillin/streptomycin) in ice. Lungs were then removed from the isolation solution and finely minced and transferred to the digestion solution (0.22 μm filtered 3 mg/mL collagenase I in DMEM) and incubated in a temperature-controlled shaker (37 °C, rpm) for 45 min. The solution was then filtered through a 70 µm nylon filter, and the collected solution was centrifuged at 1200 rpm, 4 °C for 8 min. The pellet was resuspended in PBS supplemented with 0.1% BSA and incubated with dynabeads conjugated with anti-Tie 2 antibody for 15 min at room temperature under agitation. The bead–cell complex was sorted with a magnetic rack and washed consecutively with growth media 5 times. Cells were resuspended in Vasculife^®^ VEGF-Mv (Lifeline) and grown to confluence. Cells from passages 3–6 were used in the experiments.

### 4.12. Transendothelial Electrical Resistance

Transendothelial electrical resistance (TEER) was assessed to determine the integrity of the endothelial monolayers formed by MLECs from WT and *Gpc1*^−/−^ mice. TEER was determined using the Electric Cell-substrate Impedance Sensing system ECIS Z-Theta^®^ (Applied BioPhysics, Troy, NY, USA) as previously described [50]. For each measurement, 10^5^ WT or *Gpc1*^−/−^ MLECs were plated per well in 8-well ECIS arrays in complete culture media. Cells were plated in equal confluence. TEER was monitored in real time at low AC frequency (<4000 Hz) for 24 h. Data were corrected to initial readings in each respective well (normalized TEER). Data are plotted as mean ± SEM.

### 4.13. Immunofluorescence

#### 4.13.1. Lung Tissue

Animals were anesthetized with ketamine/xylazine (90/10 mg/Kg). The thorax and abdomen were cleaned by alternating 70% ethanol and chlorhexidine 3 times. The chest was opened, and the lungs were perfused with PBS through the pulmonary artery. Lungs were perfused with 4% paraformaldehyde and kept in a fixative overnight at room temperature. Lungs were then transferred to (70%) ethanol until processing by The University of Arizona Cancer Center TACMASR Pathology Core (embedding in paraffin and sectioning). On the day of experiments, lung sections were deparaffinized, and sections were incubated with 5% normal goat serum in PBS supplemented with 0.1% Triton (PBS-T) for the blocking of non-specific sites followed by the incubation with primary antibody anti heparan sulfate (1:100 dilution in blocking buffer) overnight at 4 °C. The next day, sections were washed, blocked for 1 h with blocking buffer (5% normal goat serum in PBS-T), and then incubated with the secondary antibody (1:500 dilution in PBST) for 1 h at room temperature. Negative controls were performed by incubating lung sections with blocking buffer overnight at 4 °C. representative negative control images are shown in the Supplementary Material. The next day, sections were washed, blocked for 1 h with blocking buffer (5% normal goat serum in PBS-T), and then incubated with the secondary antibody for 1 h at room temperature. Sections were then incubated with DAPI (1 μg/mL) for 15 min, washed 3 times with PBS-T, and mounted in Prolong antifade mounting medium. Images were taken using LEICA DMI600 at The University of Arizona Imaging Core. Views were projected using the using LAS X software V. 3.7 (Leica, IL, USA).

#### 4.13.2. Mouse Lung Endothelial Cells

Mouse lung endothelial cells were plated on gelatin-coated coverslips and grown to confluence. On the day of an experiment, cells were washed with HBSS and fixed in 4%PFA for 20 min. Cells were blocked with 5% normal goat serum in PBS-T and incubated with primary antibodies (at 1:100 dilution for syndecan 1, syndecan 4, and heparan sulfate) in a wet chamber overnight at 4 °C. Negative controls were performed by incubating cells with blocking buffer overnight at 4 °C. The next day, cells were washed, blocked for 1 h with blocking buffer (5% normal goat serum in PBS-T), and then incubated with the secondary antibody (1:500 dilution in PBST) overnight at 4 °C. On the following day, cells were rinsed with PBS-T, incubated with DAPI 1µg/mL for 15 min at room temperature, washed three times for ten minutes with PBS-T, and mounted in in Prolong antifade mounting medium. Images were taken using the LEICA DMI600 microscope at The University of Arizona Imaging Core using LAS X software V. 3.7 (Leica, IL, USA); 2D, 2.5D, and 3D views were constructed using LAS X software V.1.4.5 (Leica, IL, USA). Equal brightness and contrast were applied to all images. The density of heparan sulfates, syndecan 1, and syndecan 4 in MLECs was assessed by measuring fluorescence/area using Image J software V.1.53e (developed at the U.S. National Institutes of Health), and data are shown as mean ± SEM.

### 4.14. Statistics

Statistical analyses were performed using GraphPad Prism software V. 9.2.0. Data are presented as mean ± SEM. Groups were compared using an unpaired Student’s *t*-test with Welch’s correction. For TEER experiments, two-way ANOVA with Tukey’s post hoc test was used; *p* < 0.05 was considered statistically significant for all comparisons.

## Data Availability

The data presented in this study are available from the corresponding author upon reasonable request.

## Supplementary Materials

The following supporting information can be downloaded at: https://www.mdpi.com/article/10.3390/ijms241914568/s1.
