# Supplementary material for: The Pulmonary Endothelial Glycocalyx Modifications in Glypican 1 Knockout Mice Do Not Affect Lung Endothelial Function in Physiological Conditions
Source: Int J Mol Sci. 2023 Sep 26;24(19):14568. doi: 10.3390/ijms241914568 (PMC10573009; doi:10.3390/ijms241914568)
Supplement: Supplementary file 1 [file ijms-24-14568-s001.zip › ijms-2561627-supplementary.pdf]

Supplementary Materials

The pulmonary endothelial glycocalyx modifications in glypican 1 knockout mice do not affect lung endothelial function in physiological conditions.

Lakshmi N R Thota[1], Joaquin L Rosales[1], Ivan Placencia[1], Evgeny A Zemskov [3,4] Paola Tonino [5], Ashley N. Michael [6], Stephen M Black [3,4,8] Andreia Z Chignalia[1,2,7, 9*]

Original Membranes

Western Blot

Figure 1B Lung Lysates (Representative images figure 1B in blue boxes)

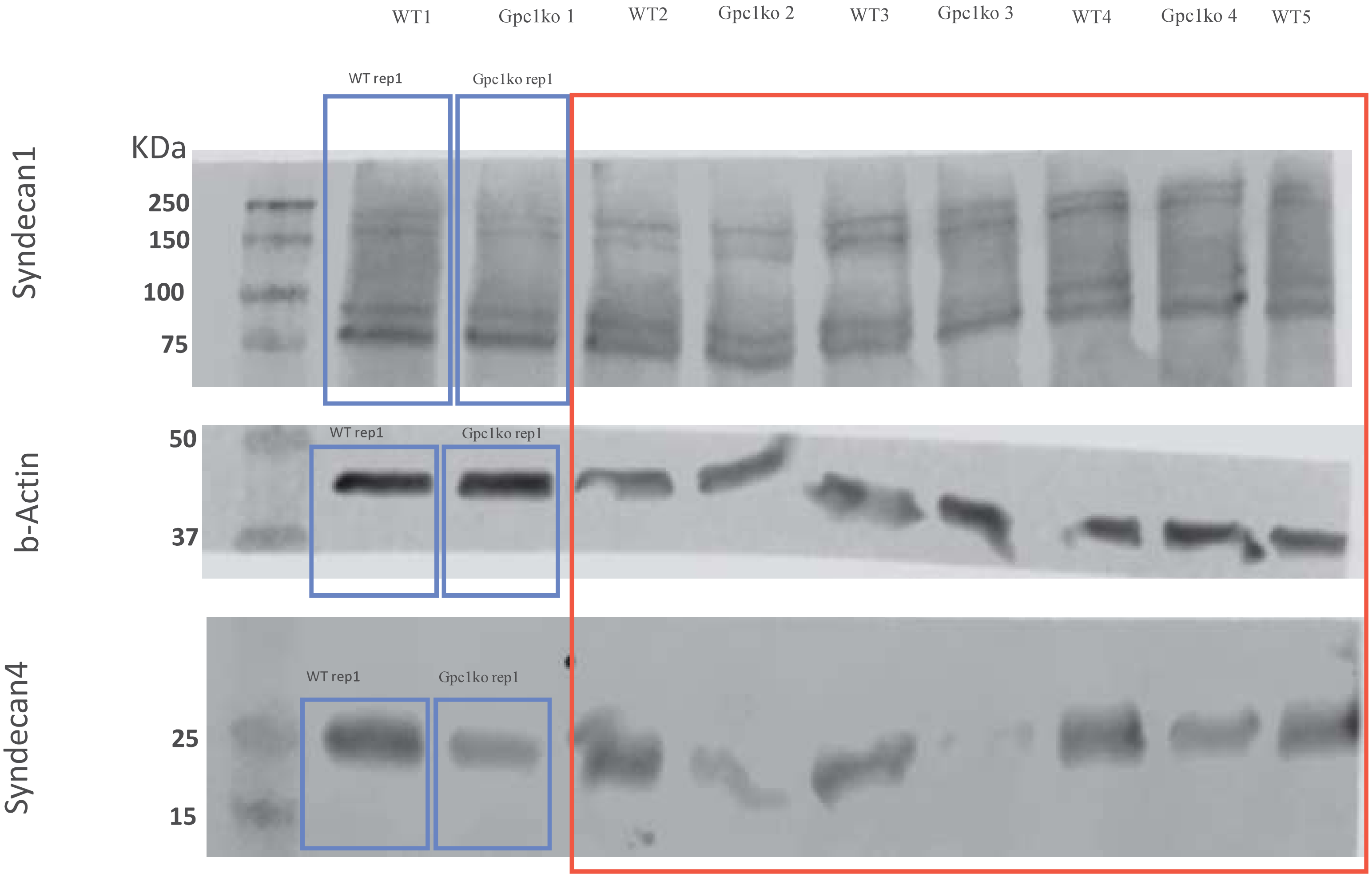

Blocking 5% BSA
Antibodies: 3% BSA

Syndecan1 Primary antibody dilution:1:1000
Secondary Ab dilution:1:5000

β-actin primary ab dilution:1:1000
Secondary Ab dilution:1:5000

Syndecan 4 primary antibody dilution:1:1000
Secondary Ab dilution:1:5000

Lanes in red boxes not quantified due to bubbles in actin band or poor gel quality

Lung Lysates (Representative images Figure 1 B in blue boxes)

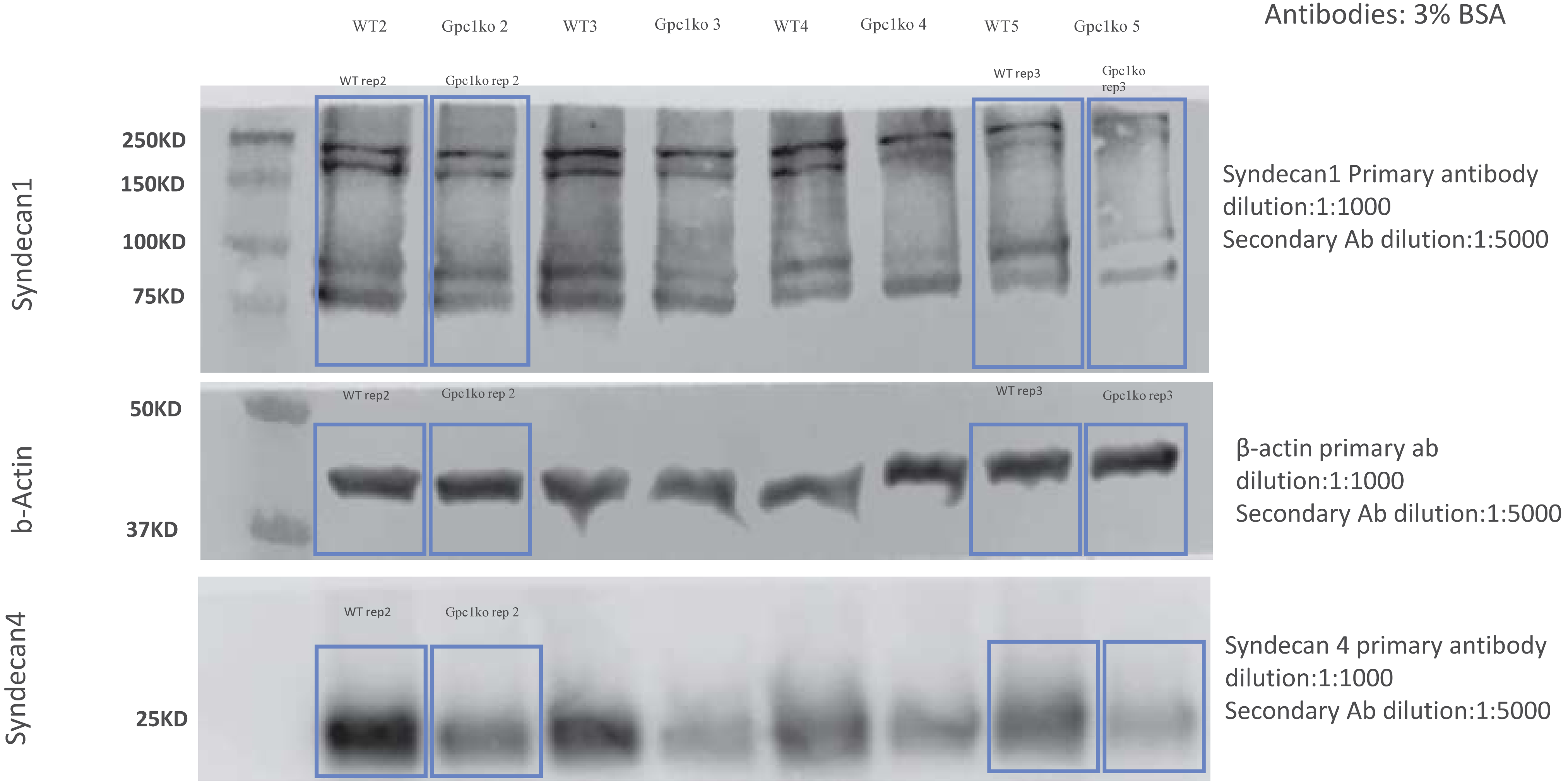

Figure 3A Lung Lysates (Representative images figure 3A)

Blocking 5% BSA
Antibodies: 3% BSA

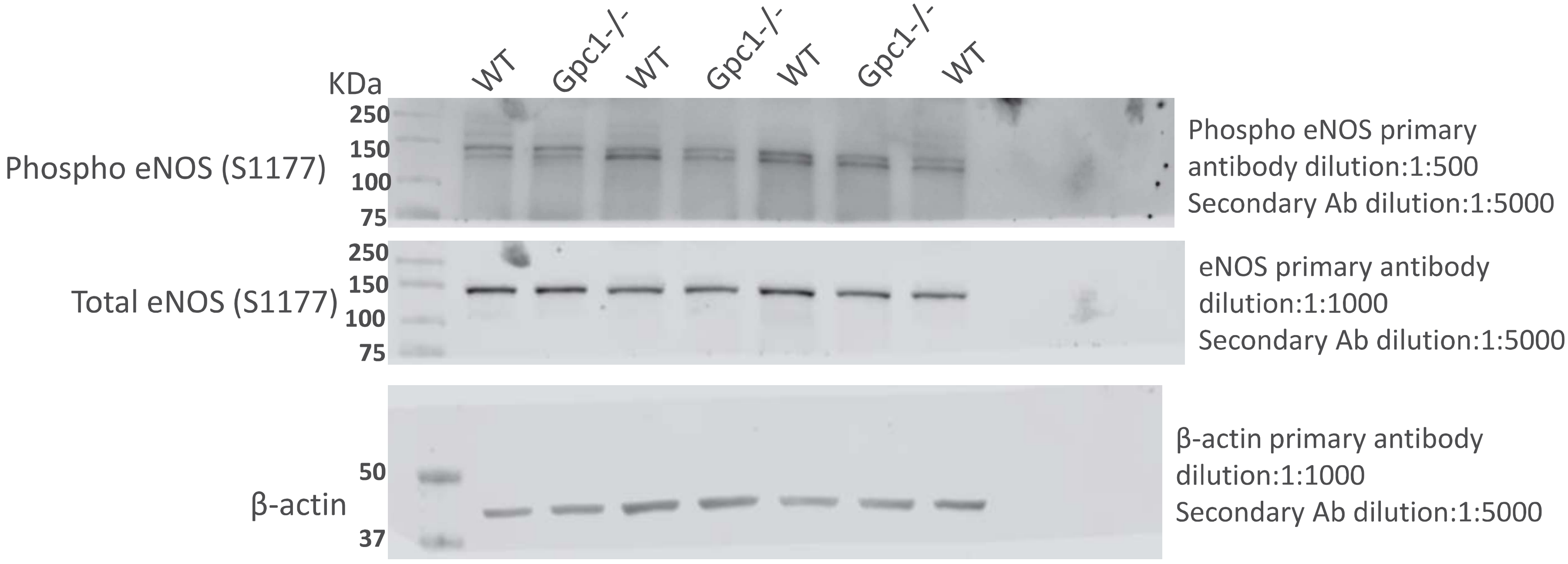

Phospho eNOS primary antibody dilution:1:500
Secondary Ab dilution:1:5000

eNOS primary antibody dilution:1:1000
Secondary Ab dilution:1:5000

β-actin primary antibody dilution:1:1000
Secondary Ab dilution:1:5000

Figure 3E Lung Lysates (Representative images figure 3E are in the blue box)

Blocking 5% BSA
Antibodies: 3% BSA

Phospho AKT primary antibody dilution:1:500
Secondary Ab dilution:1:5000

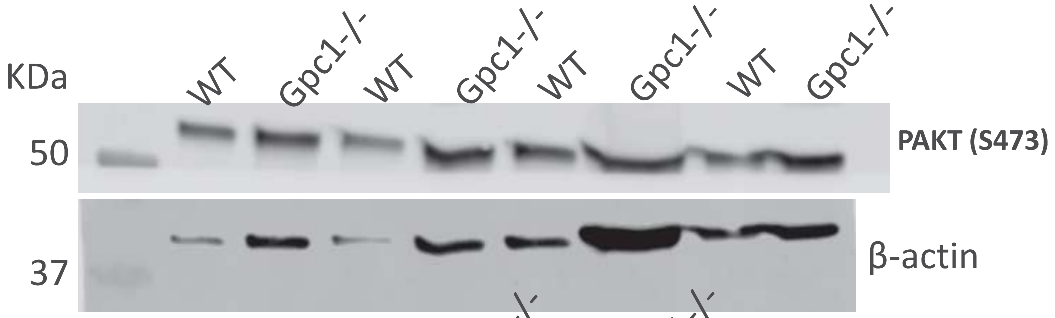

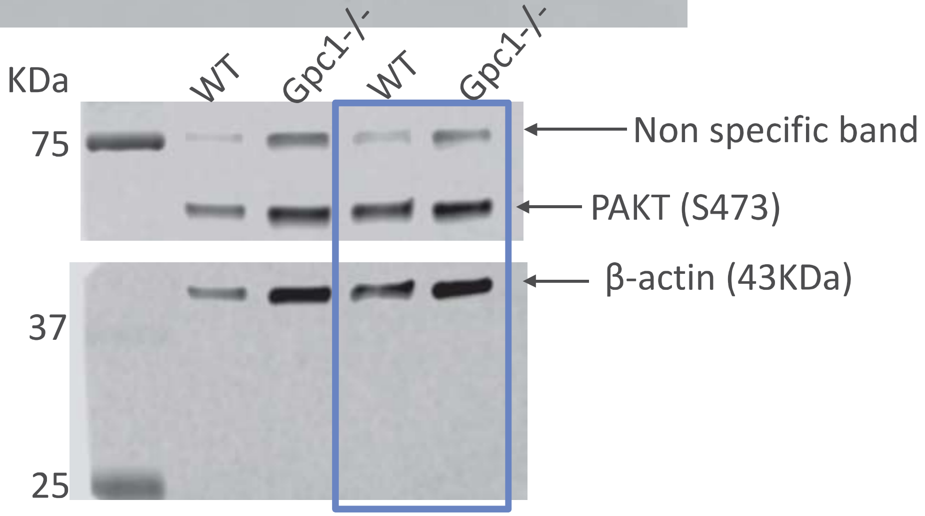

β-actin primary antibody dilution:1:1000
Secondary Ab dilution:1:5000

Supplementary material

Immunofluorescence – negative controls

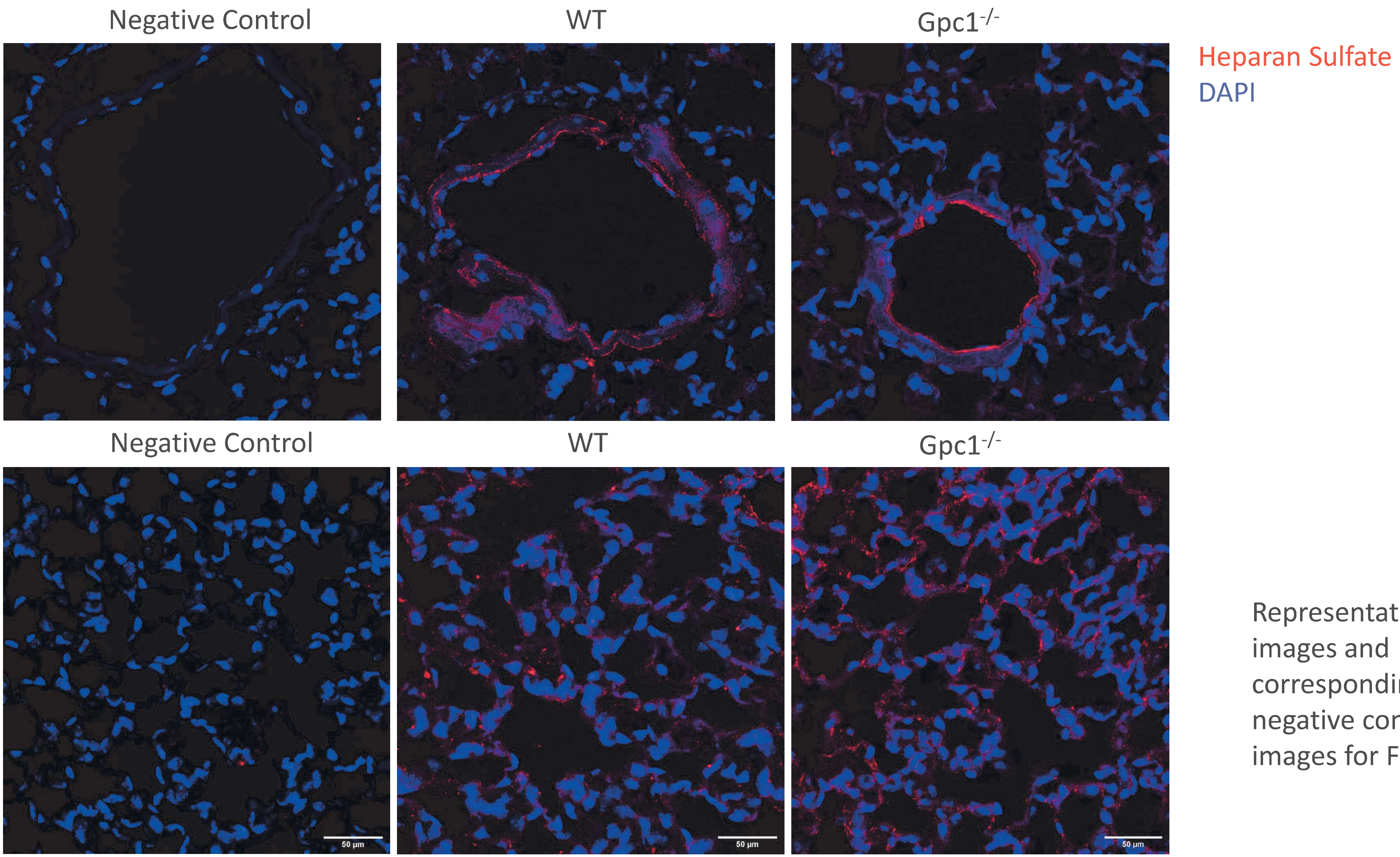

Representative images and corresponding negative control images for Figure 2

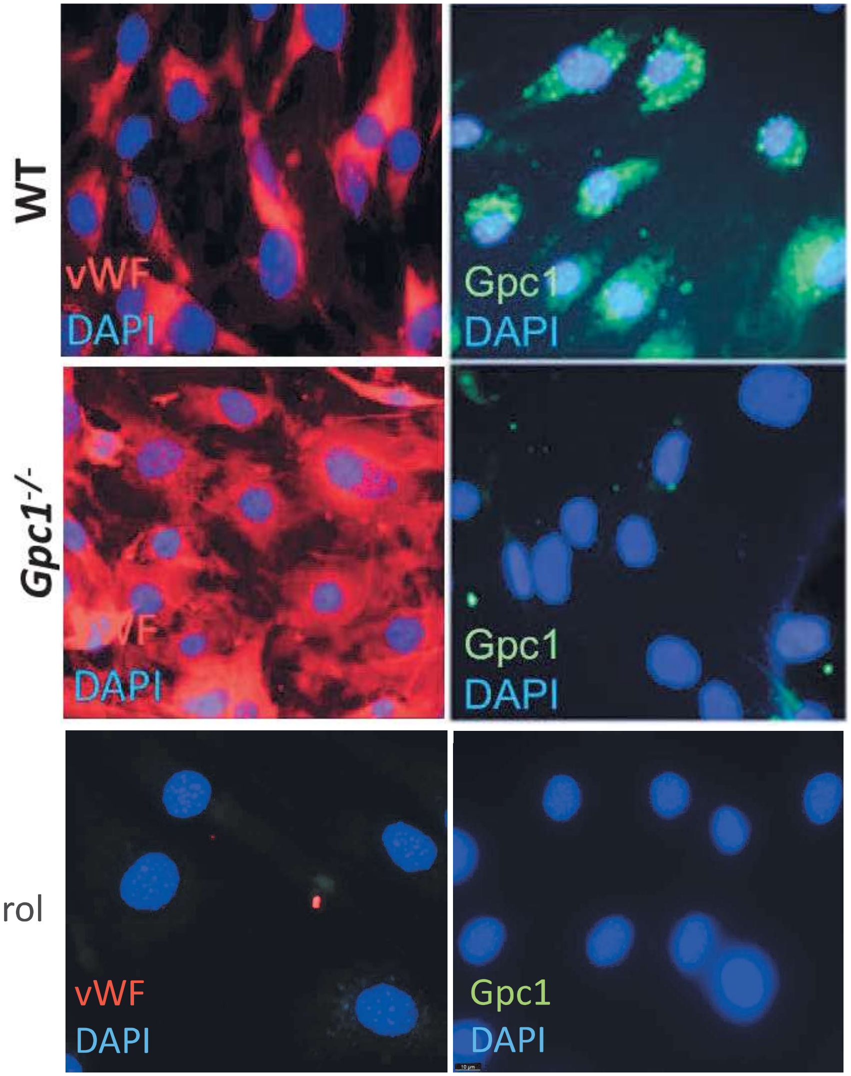

Negative Control

Representative images and corresponding negative control images for Figure 4

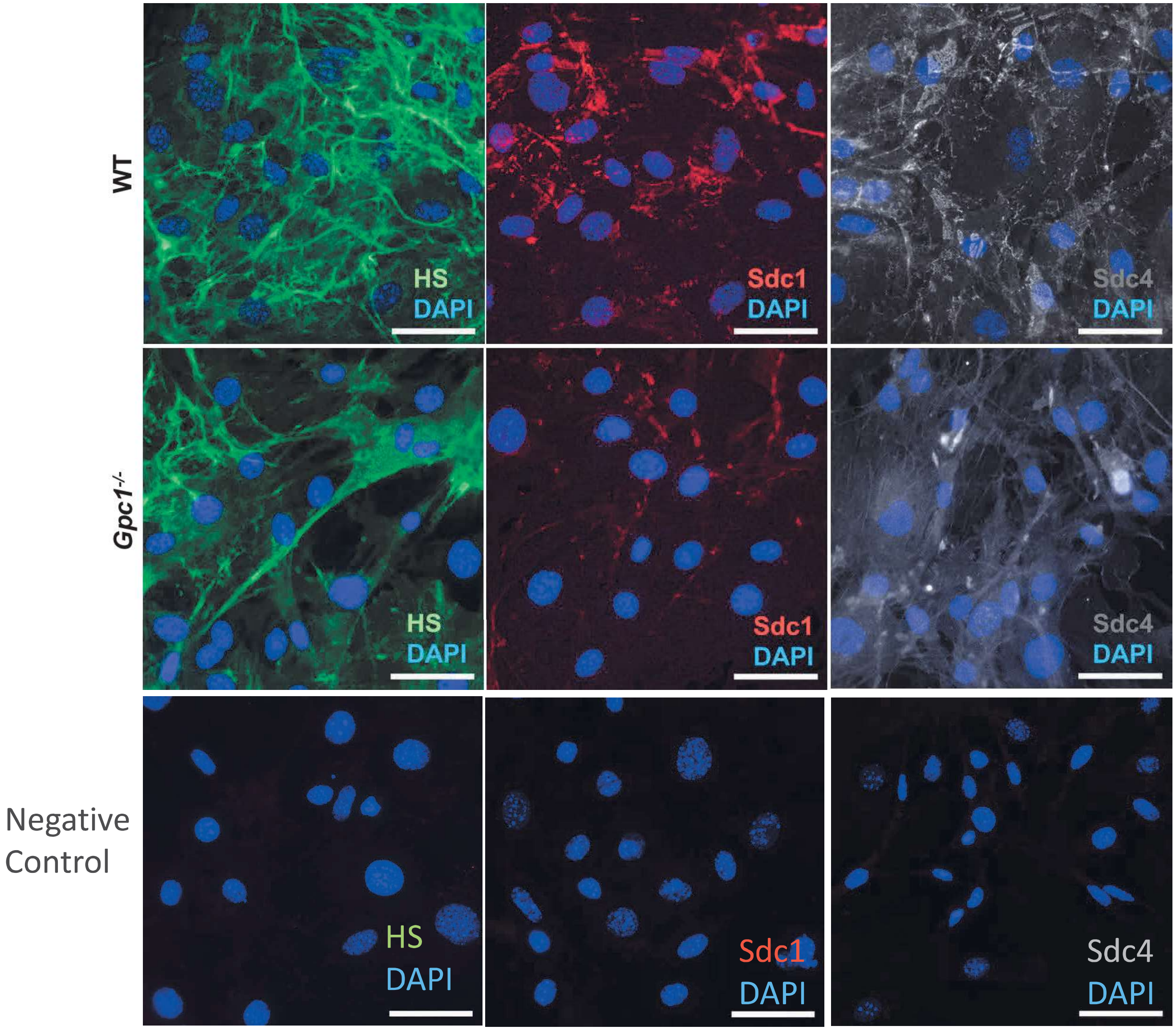

Representative images and corresponding negative control images for Figure 4
